# Customized Scleral Lenses: An Alternative Tool for Severe Dry Eye Disease—A Case Series

**DOI:** 10.3390/jcm13133935

**Published:** 2024-07-04

**Authors:** Sebastiano Nunziata, Daniele Petrini, Serena Dell’Anno, Vincenzo Barone, Marco Coassin, Antonio Di Zazzo

**Affiliations:** 1 Ophthalmology, Campus Bio-Medico University, 00128 Rome, Italy; sebastiano.nunziata@unicampus.it (S.N.); vincenzo.barone@unicampus.it (V.B.);; 2 Ophthalmology Operative Complex Unit, Campus Bio-Medico University Hospital Foundation, 00128 Rome, Italy; 3 Science Department, University of Rome III, 00154 Rome, Italy; 4 Vision Optika, 00187 Rome, Italy; optometristaserenadellanno@gmail.com; 5 Rare Corneal Diseases Center, Campus Bio-Medico University Hospital Foundation, 00128 Rome, Italy

**Keywords:** dry eye disease, age-related dry eye disease, scleral lens

## Abstract

**Purpose:** Dry eye disease (DED) is a multifactorial condition significantly impacting patients’ quality of life (QoL). This study aims to present a case series highlighting the effectiveness of customized scleral lenses in managing severe DED and improving patient outcomes. **Methods:** This case series includes three patients with severe DED refractory to conventional treatments. Customized scleral lenses were fitted for each patient, and clinical outcomes were evaluated over a period of two months. Assessments included best-corrected visual acuity (BCVA), slit-lamp examination findings, and corneal National Eye Institute (NEI) scores. **Results:** All three patients demonstrated significant improvements in BCVA, reductions in ocular symptoms, and enhanced ocular surface health. Patient 1, with secondary Sjögren’s syndrome and suspected mucous membrane pemphigoid, showed resolution of conjunctival hyperemia and epithelial defects. Patient 2, with graft versus host disease, exhibited resolution of punctate keratitis and the absence of thread mucus. Patient 3, post-oncologic surgery, achieved complete resolution of keratoconjunctivitis sicca and the restoration of vision. **Conclusions:** Customized scleral lenses are a useful therapeutic option for severe DED, providing significant symptomatic relief and enhancing patients’ quality of life. Their use should be considered in refractory cases to optimize ocular surface health and visual outcomes.

## 1. Introduction

Dry eye is a multifactorial disease of the ocular surface characterized by a loss of homeostasis of the tear film and accompanied by ocular symptoms, in which tear film instability and hyperosmolarity, ocular surface inflammation and damage, and neurosensory abnormalities play etiological roles [1,2]. Various management options include lubricating eye drops, topical corticosteroids or cyclosporine, autologous serum eye drops, punctal plugs, and minor surgical options such as punctal occlusion and tarsorrhaphy. However, constant discomfort, pain, and role limitations greatly affect patients’ quality of life [3]. Quality-of-life (QoL)-based studies have demonstrated that both mild and severe dry eye impacts QoL at a level similar to that experienced by patients with mild psoriasis and class III/IV (moderate-to-severe) angina [4]. Several studies have revealed a significant association between depression and dry eye disease (DED), showing that the prevalence of depressive symptoms among dry eye patients is higher than in other ocular disorders [5,6]. Additionally, driving skills [7], working performance [8,9], and sleep quality [10] are affected by DED. Considering the demonstrated effects of DED on life quality, its management is important to improve patient comorbidity. Clinicians should be aware of the availability of numerous therapeutic tools and their correct use. The aim of our study is to present a case series of severe dry eye disease in which customized scleral lenses were crucial in resolving refractory DED cases and improving patients’ quality of life and daily activities.

## 2. Scleral Lens

Contact lenses play an important role in the management of DED. They provide symptomatic relief and visual rehabilitation, protect the ocular surface, and keep the ocular surface moist, restoring and safeguarding what can be considered a true ecosystem of the front segment [3,11]. Several studies report their experience with the use of scleral CLs (contact lenses) in various pathologies, using different tools in terms of size and characteristics, all with excellent results for patients [12,13,14,15]. One of the most widely used and promising devices to date is PROSE (Prosthetic Replacement of the Ocular Surface Ecosystem), a device made of fluorosilicone/acrylate polymers approved in 1994 by the FDA [16]. The use of scleral lenses was limited until 1983, when a rigid gas-permeable material was introduced into their design, which resolved the common problem of hypoxia [17,18]. Throughout the 1980s and 1990s, scleral lenses were used primarily for corneal ectasia. As manufacturing capabilities and technology have expanded, the use of scleral lenses has become increasingly common, and they are now widely used for the treatment of ocular surface disease. There are several ways to categorize scleral lenses. Traditionally, scleral lenses have been classified based on diameter: semi-scleral (13.6–14.9 mm), mini-scleral (15.0–18.0 mm), and full-scleral (18.1–24.0 mm). The Scleral Lens Education Society has recently recommended a new nomenclature based on the size and fit characteristics of the lens relative to the eye. According to this system, the most important aspect of a lens is whether it rests on the cornea or the sclera, or if it touches both surfaces. A lens that rests entirely on the sclera is considered a scleral lens regardless of diameter. Regardless of the nomenclature used, there are many manufacturers and designs of scleral lenses [19].

## 3. Materials and Methods

This case series includes three patients with severe dry eye disease (DED) refractory to conventional treatments. Customized scleral lenses (MEDLAC “SLC Adapta”) were fitted for each patient for a minimum of 8 h per day, and clinical outcomes were evaluated over a two-month period. During the treatment with contact lenses, patients were allowed to use preservative-free artificial tears, applying 4–5 drops in each eye per day. Assessments included BVCA (best-corrected visual acuity), and a slit-lamp examination was performed with a Topcon DC-4 AG digital slit lamp. Images were taken with the same slit lamp. Images were obtained after fluorescein instillation, using blue cobalt and Wratten filter and 10× magnification. To evaluate interobserver concordance in measured corneal fluorescein staining (CFS), we used the National Eye Institute/Industry (NEI) grading scale. First developed in 1995, the Corneal NEI grading scale classifies the severity of corneal epitheliopathy by assigning a score from 0 to 3 to each corneal sector, depending on severity. Informed consent was obtained from every subject involved in this study.

### Customized Scleral Lenses

The MEDLAC “SLC Adapta” scleral lens is a rigid, gas-permeable contact lens. This lens is asymmetrical and free-form, technically designed to exactly replicate the profile of the ocular surface. The creation of such an “adaptive” device is made possible by the “ESP Eaglet Eye” profilometer, which provides an extensive digital impression of the ocular surface, covering both the cornea and the sclera entirely. The “SLC Adapta” is constructed within a total diameter (TD) range from a minimum of 15.80 mm to a maximum of 18.50 mm, depending on two factors: the corneal diameter and the extension of the profilometric map acquired. The lens is designed based on the actual data returned by ocular profilometry and does not utilize any artificial reconstruction of peripheral zones; rather, it faithfully and mirror-reflectively corresponds, in its entire total dimension, to the digital acquisition of the ocular surface. The inner surface of the scleral lens is divided into five zones: the central area, the optical zone—which has an average diameter of 9 mm—the haptic zone, and the scleral landing zone, which is never less than 3 mm in width. The initial limbal clearance (tear clearance at the limbus) is calibrated to an average of 150 microns, which tends to decrease, with individual variability, during subsequent wearing hours to values considered safe in the literature. The central clearance is designed to be about 200 microns with a stabilized lens after several hours of wear. The central thickness of the lens is about 250 microns, increasing to a maximum of 350 microns in the haptic zone. The material used to manufacture the SLC Adapta lenses is Optimum Infinite (Tisilfocon A III) with a DK value of 180 Iso fact. This hyper-oxygen-permeable material ensures levels of gas permeability consistently above those physiologically considered safe by the evidence regarding corneal metabolic aspects and exceptional shape stability. These lenses are always surface-treated with Hydra-Peg, a special surface coating applied by Medlac Srl (Avellino, Italy) under an international license from Tangible (San Francisco, CA, USA). This coating of PEG (polyethylene glycol) is 40-nanometers-thick and is deposited across the entire surface of the lens. The clinical purpose of Hydra-Peg is to ensure high wettability and low adhesiveness of the contact lens surface to organic lipid and mucoprotein deposits in tears, contributing to the maintenance of optical quality of vision and comfort.

## 4. Case Series: Three Patients Affected by Severe Dry Eye Disease

### 4.1. Patient 1

**Figure 1:**
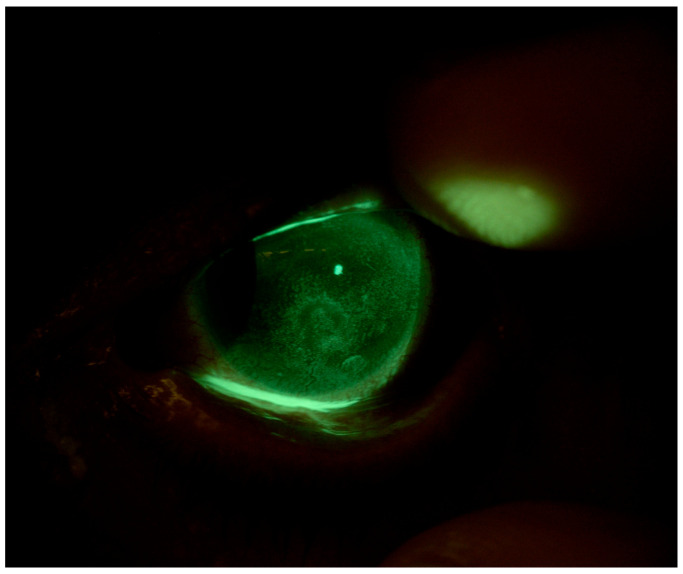
Patient 1: OS before scleral LAC application.

Patient 1 is a 78-year-old woman affected by secondary Sjögren’s syndrome undergoing therapy with hydroxychloroquine 200 mg/day and methylprednisolone 4 mg/day, with suspected mucous membrane pemphigoid (MMP). The patient complained of decreased visual acuity, pain, photophobia, and a marked increase in major conjunctival hyperemia in OS. At baseline, the patient’s BVCA (best-corrected visual acuity) was 0.22 logMAR in OD and 2.0 logMAR in OS. Slit-lamp observations revealed conjunctival hyperemia, the presence of thread mucus, reduced lacrimal meniscus, and a central epithelial defect in both eyes. The patient’s Corneal NEI score was 11 in OD and 13 in OS (Figure 1).

**Figure 2:**
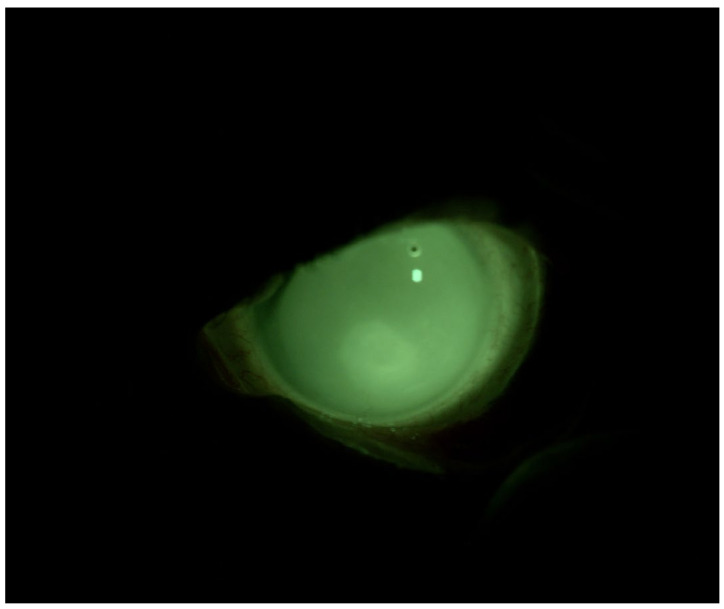
Patient 1: OS after scleral LAC application. We used a photo while the patient is wearing her CL to show the fit and the lacrimal reservoir.

The diagnosis of pemphigoid was confirmed through biopsy, and subsequently, systemic therapy with mycophenolate mofetil at a dosage of 500 mg twice daily was initiated. Following the therapeutic failure of ocular surface lubricants and topical cortisone therapy, we decided to apply the MEDLAC “SLC Adapta” for 8 h daily and keep the patient under observation. After 2 months, BVCA was 0.1 logMAR in OD and 0.22 logMAR in OS. Slit-lamp observations revealed the absence of conjunctival hyperemia, the absence of thread mucus, the resolution of the epithelial defect, and the presence of stromal opacity. The patient’s Corneal NEI score was 0 (Figure 2).

### 4.2. Patient 2

**Figure 3:**
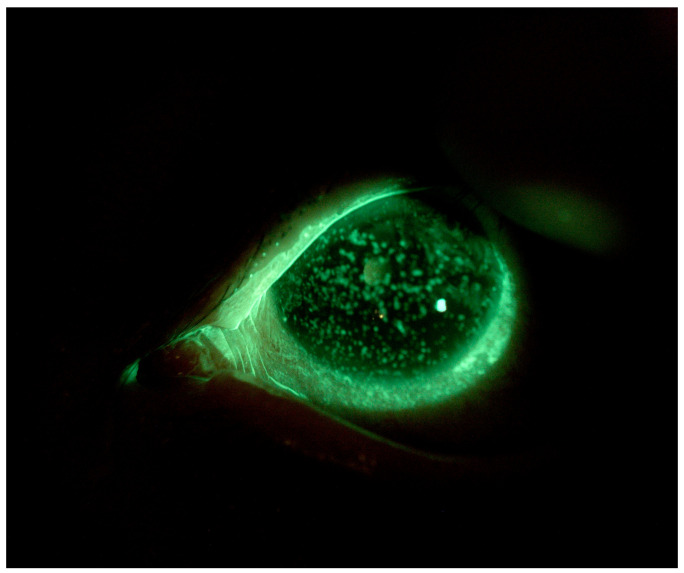
Patient 2: OS before scleral LAC application.

Patient 2 is a 48-year-old woman affected by chemo-resistant Acute Myeloid Leukemia (AML) who underwent two allogeneic stem cell transplantations followed by chronic graft-versus-host disease (GVHD) with ocular involvement. She was already on therapy with oral cyclosporine 200 mg/day, topical cyclosporine 1% (one drop three times a day), and corneal lubricants every hour. The patient complained of eye pain, itching, photophobia, and major conjunctival hyperemia, especially in OS. At baseline, the patient’s BVCA was 0.2 logMAR in both eyes. Slit-lamp observations revealed simple punctate keratitis in both eyes (OS more than OD) and the presence of thread mucus. The patient’s Corneal NEI score was 15 (Figure 3).

**Figure 4:**
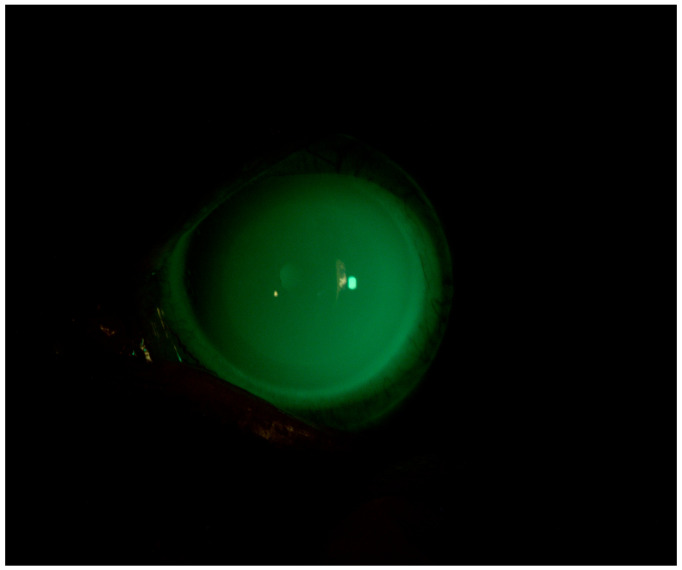
Patient 2: OS after scleral LAC application. We used a photo while the patient is wearing her CL to show the fit and the lacrimal reservoir.

Following the failure of different therapeutic adjustments with preservative-free lubricants, lubricating eye gels, and topical cortisone, we decided to apply the customized scleral lens and keep the patient under observation. After 2 months, the patient’s BVCA was 0.0 logMAR in both eyes. Slit-lamp observations revealed the resolution of simple punctate keratitis in both eyes and the absence of thread mucus. The patient’s Corneal NEI score was 0 (Figure 4).

### 4.3. Patient 3

**Figure 5:**
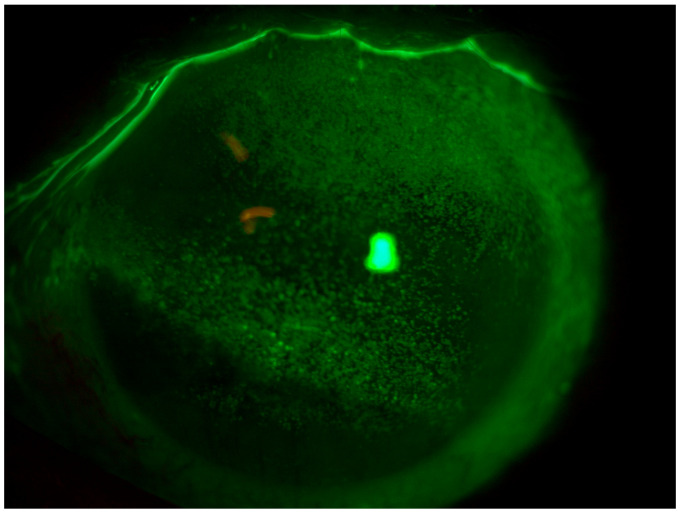
Patient 3: OS before scleral LAC application.

Patient 3 is a 53-year-old male patient who presented with complaints of eye pain, itching, photophobia, and significant conjunctival hyperemia, primarily affecting the left eye (OS). The patient had undergone oncologic surgery ten months earlier, specifically the removal of a basal cell carcinoma from the left upper eyelid. Upon clinical examination, the patient exhibited a BVCA of 0.3 logMAR in the left eye (OS). Slit-lamp examination revealed incomplete eyelid closure with a consequent significant keratoconjunctivitis sicca (KCS) involving nearly the entire corneal surface, accompanied by thread-like mucus discharge. The corneal surface showed an NEI score of 12 (Figure 5).

**Figure 6:**
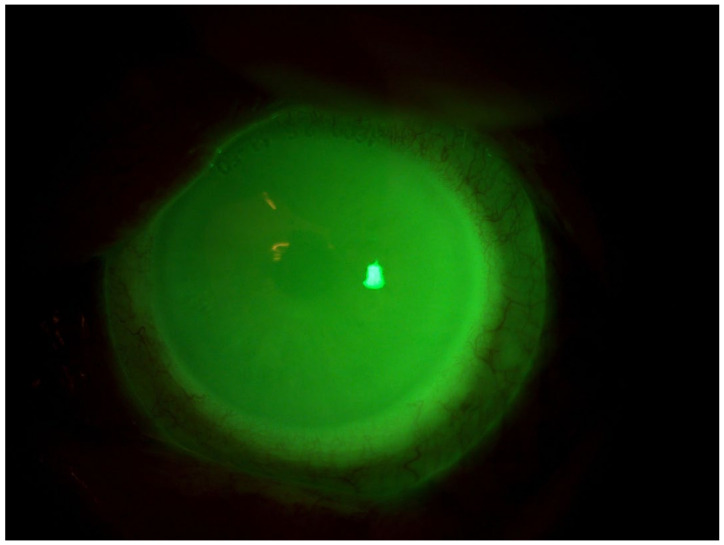
Patient 3: OS after scleral LAC application. We used a photo while the patient is wearing his CL to show the fit and the lacrimal reservoir.

Despite attempts with various therapeutic strategies involving preservative-free lubricants and overnight ointments, the patient’s symptoms persisted. Subsequently, a customized scleral lens was fitted, and nocturnal eyelid taping was initiated. After two weeks of treatment, we obtained a complete resolution of corneal surface abnormalities. Vision was fully restored to its preoperative state. During clinical assessment, the patient achieved a best-corrected visual acuity (BCVA) of 0.0 logMAR in the left eye (OS). Slit-lamp examination indicated the resolution of simple punctate keratitis and the absence of thread-like mucus in OS. The NEI score was recorded as 0 (Figure 6).

## 5. Discussion

DED severely impacts the everyday living, psychology, and overall life of patients [20], and current treatments are improving but not resolving the disease’s course [3,11]. The treatments should not only act on the symptoms but also improve the patient’s real life, work, and social activities. Custom scleral lenses could fill such a gap as an effective tool to restore the ocular surface microenvironment and improve patients’ symptoms and quality of life. Daily use of customized scleral lenses in such patients drastically changed their quality of life. The lenses reduced pain and photophobia by enhancing epithelial growth in such complex patients. Despite the many positive aspects, dry eye treatment with scleral contact lenses, like any therapy, has side effects. Visual acuity can decrease after several hours of wear, likely because of trapped debris underneath the lens and mid-term fogging [21]. Conjunctival prolapse, a condition in which loose conjunctiva folds over the cornea because of pressure from the lens, has been described, but its incidence and ramifications are unknown [22]. A review of over 300 scleral lens wearers reported corneal neovascularization (13.3% of scleral lens wearers), corneal edema (7.4%), corneal abrasion (3.1%), and giant papillary conjunctivitis (1.7%) [23,24,25,26,27,28,29,30,31,32,33,34,35,36,37]. These issues may occur despite the lenses and fitting procedures having undergone significant improvement since then [26], reducing side effects and improving comfort for patients. According to expert opinion, the issues related to scleral contact lenses, which sometimes lead to therapy discontinuation, include the need for patient training, the potential for breakage/loss, and the longer time required for customized production compared to preformed scleral contact lenses (15 days for customized scleral lenses versus 3 days for preformed scleral lenses). This case series has several limitations. Firstly, the small sample size of only three patients limits the generalizability of the findings. Larger studies are needed to confirm the efficacy of customized scleral lenses in managing severe DED across a broader population. Secondly, the follow-up period of two months may not be sufficient to fully evaluate the long-term effects and potential complications associated with the use of these lenses. Longer-term studies are necessary to assess the durability of the improvements in visual acuity and ocular surface health. Additionally, this study did not include a control group, which limits the ability to make definitive comparisons between the efficacy of customized scleral lenses.

## 6. Conclusions

Despite initial difficulties, customized scleral lenses, having a patient-based fit and a Hydra-Peg coating, showed great improvement not only in signs and symptoms but also in patients’ quality of life. With the presented results, proper patient education can make these lenses a helpful tool, especially in severe dry eye disease. Future clinical trials would be necessary to compare them with other widely used therapies for dry eye in patients refractory to standard treatments.

## Data Availability

Due to privacy concerns, the data utilized in this study are not publicly available.
